# A Coagulation-Related Gene-Based Prognostic Model for Invasive Ductal Carcinoma

**DOI:** 10.3389/fgene.2021.722992

**Published:** 2021-09-21

**Authors:** Jing Li, Jiajia Du, Yanhong Wang, Hongyan Jia

**Affiliations:** ^1^Department of Breast Surgery, First Hospital of Shanxi Medical University, Taiyuan, China; ^2^Department of Microbiology and Immunology, Shanxi Medical University, Taiyuan, China

**Keywords:** invasive ductal carcinoma, coagulation, SERPINA1, HMGCS2, MMP7, plat, prognosis

## Abstract

**Background:** Invasive ductal carcinoma (IDC) is the most common type of metastatic breast cancer. Due to the lack of valuable molecular biomarkers, the diagnosis and prognosis of IDC remain a challenge. A large number of studies have confirmed that coagulation is positively correlated with angiogenesis-related factors in metastatic breast cancer. Therefore, the purpose of this study was to construct a COAGULATION-related genes signature for IDC using the bioinformatics approaches.

**Methods:** The 50 hallmark gene sets were obtained from the molecular signature database (MsigDB) to conduct Gene Set Variation Analysis (GSVA). Gene Set Enrichment Analysis (GSEA) was applied to analyze the enrichment of HALLMARK_COAGULATION. The COAGULATION-related genes were extracted from the gene set. Then, Limma Package was used to identify the differentially expressed COAGULATION-related genes (DECGs) between ductal carcinoma *in situ* (DCIS) and invasive ductal carcinoma (IDC) samples in GSE26340 data set. A total of 740 IDC samples from The Cancer Genome Atlas (TCGA) database were divided into a training set and a validation set (7:3). The univariate and multivariate Cox regression analyses were performed to construct a risk signature, which divided the IDC samples into the high- and low-risk groups. The overall survival (OS) curve and receiver operating characteristic (ROC) curve were drawn in both training set and validation set. Finally, a nomogram was constructed to predict the 1-, 2-, 3-, 4-, and 5-year survival rates of IDC patients. Quantitative real-time fluorescence PCR (qRT-PCR) was performed to verify the expression levels of the prognostic genes.

**Results:** The “HALLMARK_COAGULATION” was significantly activated in IDC. There was a significant difference in the clinicopathological parameters between the DCIS and IDC patients. Twenty-four DECGs were identified, of which five genes (*SERPINA1*, *CAPN2*, *HMGCS2*, *MMP7*, and *PLAT*) were screened to construct the prognostic model. The high-risk group showed a significantly lower survival rate than the low-risk group both in the training set and validation set (*p*=3.5943e-06 and *p*=0.014243). The risk score was demonstrated to be an independent predictor of IDC prognosis. A nomogram including risk score, pathological_stage, and pathological_N provided a quantitative method to predict the survival probability of 1-, 2-, 3-, 4-, and 5-year in IDC patients. The results of decision curve analysis (DCA) further demonstrated that the nomogram had a high potential for clinical utility.

**Conclusion:** This study established a COAGULATION-related gene signature and showed its prognostic value in IDC through a comprehensive bioinformatics analysis, which may provide a potential new prognostic mean for patients with IDC.

## Introduction

According to the GLOBOCAN 2020 cancer statistics, breast cancer (BRCA) has become one of the most common malignancies worldwide with an incidence and mortality rate of 47.8 per 100,000 and 13.6 per 100,000, respectively, and patients with breast cancer account for the highest proportion of cancers in women (Sung et al., 2021). Breast cancer, as a highly heterogeneous tumor, has different phenotypes and biological behaviors due to differences in genomics, transcriptomics, and tumor microenvironmental, thus showing different morphological manifestations, treatment response, and histological grading (Rakha et al., 2010). Among breast cancer, invasive ductal carcinoma (IDC) is the most common tissue type that accounts for 75–80% of the disease (Low et al., 2018). Compared to ductal carcinoma *in situ* (DCIS), IDC cells can break through the basement membrane and can metastasize to distant sites, and the high metastatic and invasive capacity of IDC is the main cause of death in breast cancer patients. The invasion and metastasis of cancer is a complex process controlled by various molecular determinants and may involve activation of oncogenes, inactivation of tumor suppressor genes, and aberration of the related signaling pathways (Fan et al., 2014; Deng et al., 2019). Therefore, uncovering the key molecular biomarkers or targets involved in breast cancer invasion and metastasis may help develop novel strategies for the diagnosis and treatment of IDC.

The main molecular biomarkers currently used in the diagnosis and treatment of breast cancer include estrogen receptor (ER), progesterone receptor (PR), cell proliferation antigen Ki67, and human epidermal growth factor receptor 2 (HER-2). These prognostic biomarkers can reflect tumor growth pattern, growth rate, malignant phenotype, recurrence, and metastasis and may often inform the prognosis of breast cancer and guide endocrine therapy and chemotherapy (Dunnwald et al., 2007; Kontzoglou et al., 2013; Burstein, 2020). However, the expression status of these traditional prognostic markers can only be obtained by biopsy puncture or postoperative pathology, and some triple-negative breast cancer patients have a higher rate of tumor recurrence and metastasis and a worse prognosis due to the lack of clear therapeutic targets. Therefore, it is necessary to discover new, easy, and convenient prognostic markers for patients with IDC (Yin et al., 2020).

The coagulation system, especially the tissue factor (TF) pathway, is an important innate defense mechanism (Khorana et al., 2019), and tumor cells use the function of these host-protective pathways to shape the tumor microenvironment (TME) and to metastasize. A large amount of experimental evidence shows that the organism of malignant tumor patients is in a hypercoagulable and hyperfibrinolytic state (a state that is caused by enhanced coagulation and massive fibrin production in the early stages of the disease), and the degree of coagulation disorders correlates with tumor invasion, metastasis and patient prognosis (Tas et al., 2013b). Inhibiting the coagulation cascade at the three stages of formation of prothrombin activator, thrombin, and fibrin (Ruf and Mueller, 2006) and blocking selective platelet activation pathways (Haemmerle et al., 2018) can prevent the spread of metastatic tumors. Many coagulation disorder-related biomarkers have shown significant prognostic relevance in various cancers (Tas et al., 2013a; Marfia et al., 2016; Ji et al., 2018; Li et al., 2019; Zhang et al., 2020).

Tissue factor (TF), an integral membrane protein, is the primary initiator of the extrinsic clotting pathway. TF promotes tumor growth, migration, and angiogenesis, independent of the coagulation cascade, *via* activation of protease-activated receptor (PAR)2 (Ruf et al., 2010). Some studies found that TF can mediate microvesicular shedding of MDA-MB-231 breast cancer cells and participate in cancer cell invasion and metastasis (Rondon et al., 2018). The activation of TF and thrombin in carcinoma *in situ* of the breast is an invasive phenomenon that promotes tumor cell infiltration and metastasis and can be used to predict the prognosis of breast cancer (Shaker et al., 2020). D-dimer (DD), as a coagulation indicator, is a product of fibrin degradation. DD is increased in breast cancer, with raised levels being found in 58% of patients with involved lymph nodes and only 8% of patients without lymph node disease (Blackwell et al., 2000). Circulating tumor cells (CTC) are associated with a hypercoagulable blood state in patients with metastatic breast cancer (Kirwan et al., 2020). Moreover, DD levels are closely related to the ability of tumor patients to tolerate chemotherapy (Yuan et al., 2017) and to treatment response (Louneva et al., 2020). Thrombin-antithrombin III (TAT) complex plasma concentration is used to assess the activation of the coagulation system as a surrogate marker for activated thrombin (Blanke et al., 1987). Plasma TAT and DD are higher in early breast cancer patients than in healthy controls (Topcu et al., 2015). Circulating fibrinogen, which is converted to fibrin by thrombin, has shown utility in distinguishing breast cancer from benign breast disease (Fu et al., 2017) and increases with disease progression (Edwards et al., 1987). In addition, high tissue expression levels of urokinase-type plasminogen activator (uPA) and plasminogen activator inhibitor-1 (PAI-1) in the fibrinolytic system are also prognostic factors in breast cancer and are associated with recurrence and metastasis (Grøndahl-Hansen et al., 1993). Therefore, we hypothesized that coagulation-related biomarkers play a crucial role in the assessment of breast cancer prognosis. To our knowledge, studies on coagulation-related genes and prognosis in invasive ductal carcinoma have rarely been reported.

In this study, we systematically evaluated the expression profile, prognostic significance, and role of COAGULATION-related genes by integrating data from the GEO database, TCGA database, and the GSEA database. Based on our analysis of those data, we constructed a prognostic model that may be used to predict individualized outcomes for patients with IDC.

## Materials and Methods

### Acquisition of Data Sets and Human Tumor Samples

Fifty hallmark gene sets were downloaded from Molecular signature database (MsigDB), and the raw chip data of GSE26340 data set, which included 31 ductal carcinoma *in situ* (DCIS) samples and 36 infiltrative ductal carcinoma (IDC) samples, were downloaded from the Gene Expression Omnibus (GEO) database.^1^ The IDC transcriptome and clinical data information were downloaded from The Cancer Genome Atlas (TCGA)^2^ that includes 740 IDC tissues.

All human breast cancer tissue samples used in the study were obtained from patients who were operated on in the First Affiliated Hospital of Shanxi Medical University, from 2019 to 2021. The tissues included 5 ductal carcinoma *in situ* (DCIS) samples and 10 infiltrative ductal carcinoma (IDC) samples. All tissue samples were evaluated by two experienced pathologists. This study was approved by the Medical Ethics Committee of the First Hospital of Shanxi Medical University and was performed under the approved guidelines. Informed consents were acquired from each breast cancer patient. The tumor tissue was immediately frozen and stored at −80°C.

### Gene Set Variation Analysis and Gene Set Enrichment Analysis

We divided samples from the GSE26034 data set into the DCIS and IDC groups. Using the hallmark gene sets as the reference gene set and setting the *p*-value to <0.05 and the|t value|>2 as the cutoff criteria, we performed GSVA between DCIS and IDC patients by using the GSVA package 1.38 in R (Hänzelmann et al., 2013). The common activation/inhibition pathways of IDC were determined. GSEA was performed to analyze the difference between DCIS and IDC patients *via* javaGSEA 4.1.0 to show the HALLMARK_COAGULATION plot (Mootha et al., 2003; Subramanian et al., 2005).

### Comparison Analysis of Clinical Characteristics Between DCIS and IDC Subtypes in the GSE26034 Data Set

The difference in each clinicopathological characteristic between the DCIS and IDC subtypes was analyzed by the chi-square test.

### Differentially Expressed Analysis and Correlation of Analysis of COAGULATION-Related Gene

COAGULATION-related genes were extracted from the gene set. By using the Screening conditions of|log_2_FC|>0.5, value of *p*<0.05, the differentially expressed COAGULATION-related genes (DECGs) were determined between DCIS and IDC samples in the GSE26304 data set were analyzed using Limma Package, and then, the heat map showing the expression of DECGs was drawn *via* “heatmap 1.0.12” in R.^3^ In addition, Pearson correlation analysis was conducted between these DECGs. Pearson correlation coefficient (r)>0.6 and a value of *p*<0.05 were considered as medium strong correlations.

### Construction of a Prognostic COAGULATION-Related Gene Signature Using Univariate and Stepwise Multivariate Cox Regression Analyses

Combined with the clinical information of IDC patients in the TCGA database, the genes related to the overall survival (OS) of IDC patients were screened by univariate Cox regression analysis *via* “survival 3.2-7” package in R, and *p*<0.05 was used as the criteria of selection. Then, the genes with value of *p*<0.05 were used for stepwise multivariate Cox regression analysis to construct a risk model. “Forestplot” package 1.10 in R was used to generate a forest map to show the HR (hazard ratio), lower/upper 95% CI, and *p*-value.^4^

### Estimation of the COAGULATION-Related Gene Signature for IDC Prognosis

According to the “survival” package in R and linear model, the predict. Coxph function was used to calculate the risk score of each IDC patient in the training set and validation set. The formula is as follows:

$$\mathrm{Riskscore}=h_{0}{\left(t\right)}^{*}\exp.\left(\beta_{1}X_{1}+\beta_{2}\mathrm{X2}+\ldots +\beta_{n}X_{n}\right)$$

where β is the coefficient and X is the normalized expression of each signature gene.

Then, we drew the corresponding heat map and risk curve. To determine whether the risk model has prognostic value, the Kaplan–Meier survival curves of high- and low-risk groups were drawn *via* the “survminer 0.4.8” package in R.^5^ In addition, we drew the survival curves of each gene in training set and validation set, respectively. ROC curves were drawn using the R package “survival ROC 1.0.3” for assessing and validating the effectiveness of the risk model. The area under curve (AUC) values of 1-, 2-, 3-, 4-, and 5-year survival were calculated.

### Independence of the Prognostic Model From Other Clinicopathological Characteristics

To determine whether the prognostic model is independent in other clinical characteristics, all clinicopathological characteristics in the TCGA-IDC data set, such as age, gender, pathological tumor stage, pathological N stage, pathological T stage, pathological M stage, molecular subtypes, and treatment, were included for analysis. To determine which factors have the independent prognostic value, we performed univariate and multivariate Cox regression analyses on these clinical characteristics and risk scores. The factors with *p*<0.05 were considered to have the independent prognostic value.

### Construction and Verification of the Survival Prediction Nomogram

Based on the independent prognostic analysis, we found that the prognostic model risk score, pathological_stage, Pharmaceutical_Therapy, and Both_Treatment were the independent prognostic factors that could be utilized to predict the survival rate in the TCGA-IDC data set. Therefore, the four independent prognostic factors and the molecular subtypes of IDC were used to build the nomogram *via* R package “rms 6.0–1.^6^” Besides, the survival ROC curve of these factors was used to validate the nomogram. Furthermore, the decision curve analysis (DCA), which can assess and compare prediction models that incorporate clinical consequences, was used to determine whether the nomogram was suitable for the clinical application. The x-axis indicates the percentage of threshold probability, and the y-axis represents the net benefit.

### qRT-PCR Analysis

Total RNA was extracted from the tissues of infiltrative ductal carcinoma and ductal carcinoma *in situ* tissues using Nuclezol LS RNA Isolation Reagent Kit (ABP Biosciences Inc) following the manufacturer’s instructions.

Subsequently, cDNA was synthesized from total RNA using SureScript-First-strand-cDNA-synthesis-kit (GeneCopoeia, China). Then, RT-qPCR was performed using BlazeTaq™ SYBR ® Green qPCR Mix 2.0 (GeneCopoeia, China) on a CFX96 real-time quantitative fluorescence PCR instrument (Bio-Rad, United States) according to the following protocol: pre-denaturation at 95°C for 30s, denaturation at 95°C for 10s, annealing at 60°C for 20s, and extension at 72°C for 30s, and the whole reaction cycle 40 times.

The primers used were as follows: PLAT: fw: 5'-GCCGTGAATTTAAGGGACGC, rev: 3'-GCTCGCTGCAACCTTGGTAA, SERPINA1: fw: 5'-CCTTCACTGTCAACTTCGGG, rev: 3'-TGTGTCTCTGTCAAGCTCCT, HMGCS2: fw: 5'-TCTCTTATGGCTCTGGTTTAG, rev: 3'-GCTCTCTTTGGTTCATTATTT, MMP7: fw: 5'-TGACTCAGAAACAAAAAATGCC, rev: 3'-TACGATCCTGTAGGTGACCACT, and Gapdh: fw: 5'-CGCTGAGTACGTCGTGGAGTC, rev: 3'-GCTGATGATCTTGAGGCTGTTGTC. For each sample, qPCR assays were conducted in triplicate in a 10ml reaction volume. Using the 2−ΔΔCt method, we analyzed the RT-qPCR data by normalizing gene expression to GAPDH expression as an endogenous control.

### Statistical Analysis

All the statistical analyses were performed using R software 4.0.3. Statistical significance was set as probability value *p*<0.05. The chi-square test was used to analyze the differences between the DCIS/IDC subtypes and clinicopathological parameters. The Kaplan–Meier survival curves were built to analyze the survival differences between the high-risk and low-risk groups. Univariate and multivariate Cox proportional hazard models were generated to estimate the hazard ratios of prognostic factors and to select independent prognostic factors. The ROC curves and DCA were compared to determine the predictive accuracy of the independent prognostic factors.

## Results

### “HALLMARK_COAGULATION” Pathway Is Hyperactivated in IDC Patients

**Figure 1:**
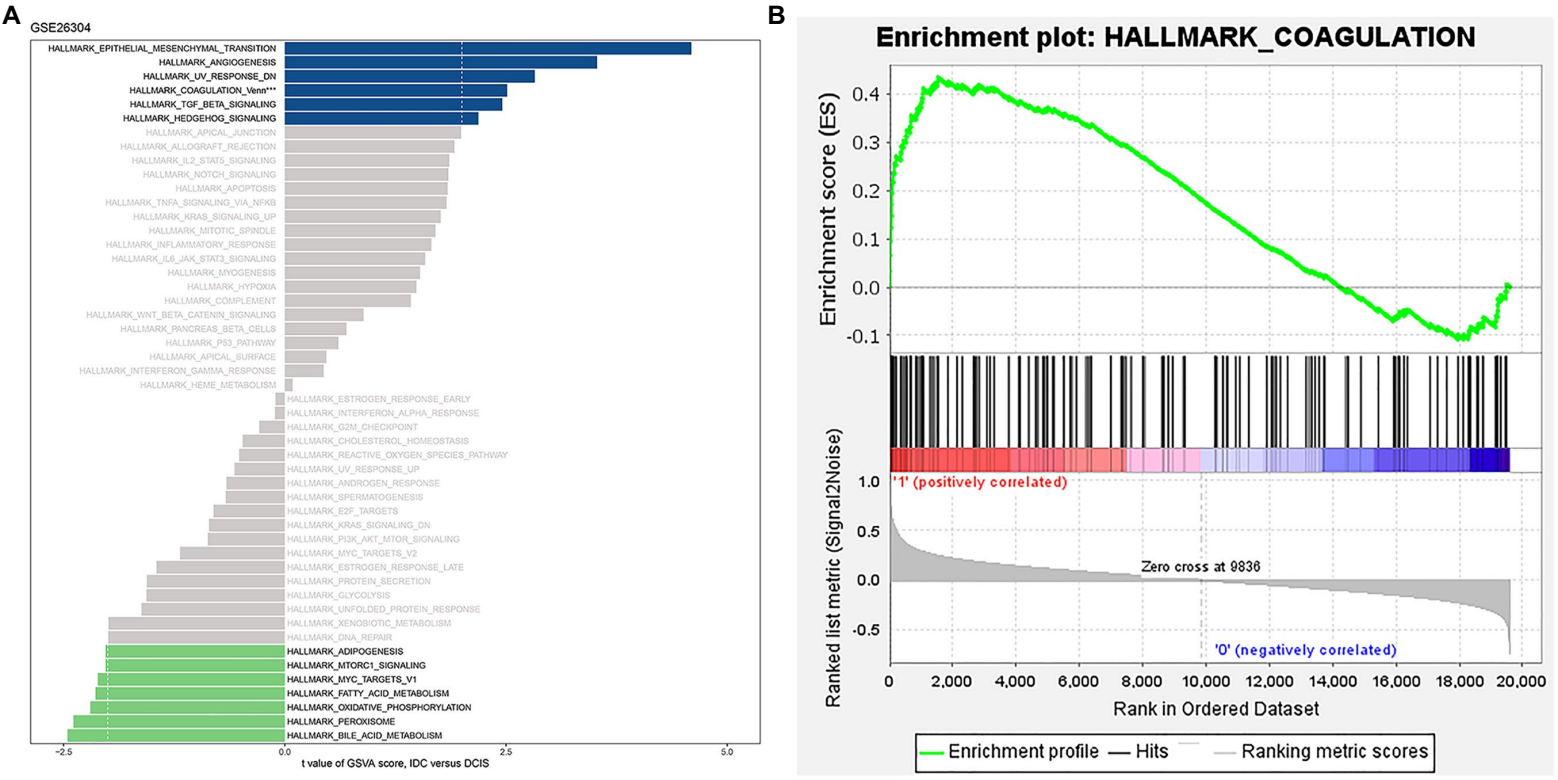
“HALLMARK_COAGULATION” pathway was significantly activated in IDC. **(A)** GSVA of the 50 hallmark gene sets in the GSE26034 data set. **(B)** Enrichment plot of “HALLMARK_COAGULATION” pathway.

We conducted GSVA of hallmark gene sets from the GSE26304 data set. The results demonstrated that the “HALLMARK_EPITHELIAL_MESENCHYMAL_TRANSITION,” “HALLMARK_ANGIOGENESIS,” “HALLMARK_UV_RESPONSE_DN,” “HALLMARK_TGF_BETA_SIGNALING,” “HALLMARK_HEDGEHOG_SIGNALING”_and “HALLMARK_COAGULATION” pathways with t value >2 were significantly activated in IDC (Figure 1A). Previous studies have revealed the correlations between these signaling pathways. Epithelial-to-mesenchymal transition (EMT) and hedgehog signaling pathways can lead to angiogenesis, loss of normal organ function, or cancer by modulating TGF-β signaling pathway in cancer progression (Wang et al., 2013; Bausch et al., 2020; Sha et al., 2020). In skin diseases, the promoted angiogenesis and lymphatic vessel enlargement were a response to ultraviolet (UV; Okazaki et al., 2012). Moreover, angiogenesis was decreased by inhibiting coagulation (Nadir, 2019). EMT also promotes basal mammary stem cell and tumor-initiating cell stemness by inducing hedgehog signaling (Guen et al., 2017). The inhibition of hedgehog signaling attenuates UVB-induced skin photoaging, reducing blood coagulation (Kim et al., 2015; Shahramian et al., 2019). CTCs expressing EMT traits could harbor enhanced procoagulant activity that could facilitate early metastasis (Bourcy et al., 2016). Hao et al. (2019) have revealed that TGF-β induces EMT to promote tumorigenesis. The inhibition of TGF-β by UV disrupted immune homeostasis in systemic lupus erythematosus (SLE; Rekik et al., 2018). In addition, several situations reported the cross talks between the TGF-β and hedgehog signaling pathways (Pelullo et al., 2019). Then, we performed GSEA of the “HALLMARK_COAGULATION” gene sets and found that it was strikingly positively enriched in IDC samples (Figure 1B). These results suggest that COAGULATION-related genes might be involved in the pathogenesis of IDC.

### Differences in Clinical Characteristics Between DCIS and IDC Subtypes

We analyzed the differences in clinical characteristics between IDC and DCIS patients. And, there were significant differences in the clinicopathological parameters between DCIS and IDC patients, especially grade (*p*=0.00209), tumor size (*p*<0.001), X-ray treatment (*p*=0.024), and hormone treatment (*p*=0.00454; Table 1). In consideration of the significant differences in clinical characteristics between IDC/DCIS and the significant enrichment of the “HALLMARK_COAGULATION” pathway in IDC, we further performed the prognostic analysis of COAGULATION-related genes in IDC.

**Table 1:** Statistical analysis of clinical characteristics of IDC/DCIS patients in the GSE26304 data set.

Samples					*p*-value
	Total (*N*=67)	DCIS (*N*=31)	IDC (*N*=36)
tp53mut				
tp53_WT	58 (86.6%)	26 (83.9%)	32 (88.9%)	0.809
tp53_Mut	9 (13.4%)	5 (16.1%)	4 (11.1%)	
Age (years)				
<=55	28 (41.8%)	14 (45.2%)	14 (38.9%)	0.787
>55	39 (58.2%)	17 (54.8%)	22 (61.1%)	
Grade				
1	3 (4.5%)	2 (6.5%)	1 (2.8%)	0.00209
2	41 (61.2%)	12 (38.7%)	29 (80.6%)	
3	23 (34.3%)	17 (54.8%)	6 (16.7%)	
Tumor Size				
Large	27 (40.3%)	26 (83.9%)	1 (2.8%)	<0.001
Small	40 (59.7%)	5 (16.1%)	35 (97.2%)	
X-ray				
1	39 (58.2%)	13 (41.9%)	26 (72.2%)	0.024
2	28 (41.8%)	18 (58.1%)	10 (27.8%)	
Chemotherapy				
1	2 (3.0%)	0 (0%)	2 (5.6%)	0.54
2	65 (97.0%)	31 (100%)	34 (94.4%)	
Hormone treatment				
1	10 (14.9%)	0 (0%)	10 (27.8%)	0.00454
2	57 (85.1%)	31 (100%)	26 (72.2%)	
Pam50 Class				
BASAL	12 (17.9%)	6 (19.4%)	6 (16.7%)	0.648
HER 2	7 (10.4%)	2 (6.5%)	5 (13.9%)	
LUMINALA	15 (22.4%)	7 (22.6%)	8 (22.2%)	
LUMINAL B	18 (26.9%)	7 (22.6%)	11 (30.6%)	
NORMAL	15 (22.4%)	9 (29.0%)	6 (16.7%)	

Significance: 1 represents No, 2 represents Yes.

### COAGULATION-Related Genes Expression and Correlation Analysis

**Figure 2:**
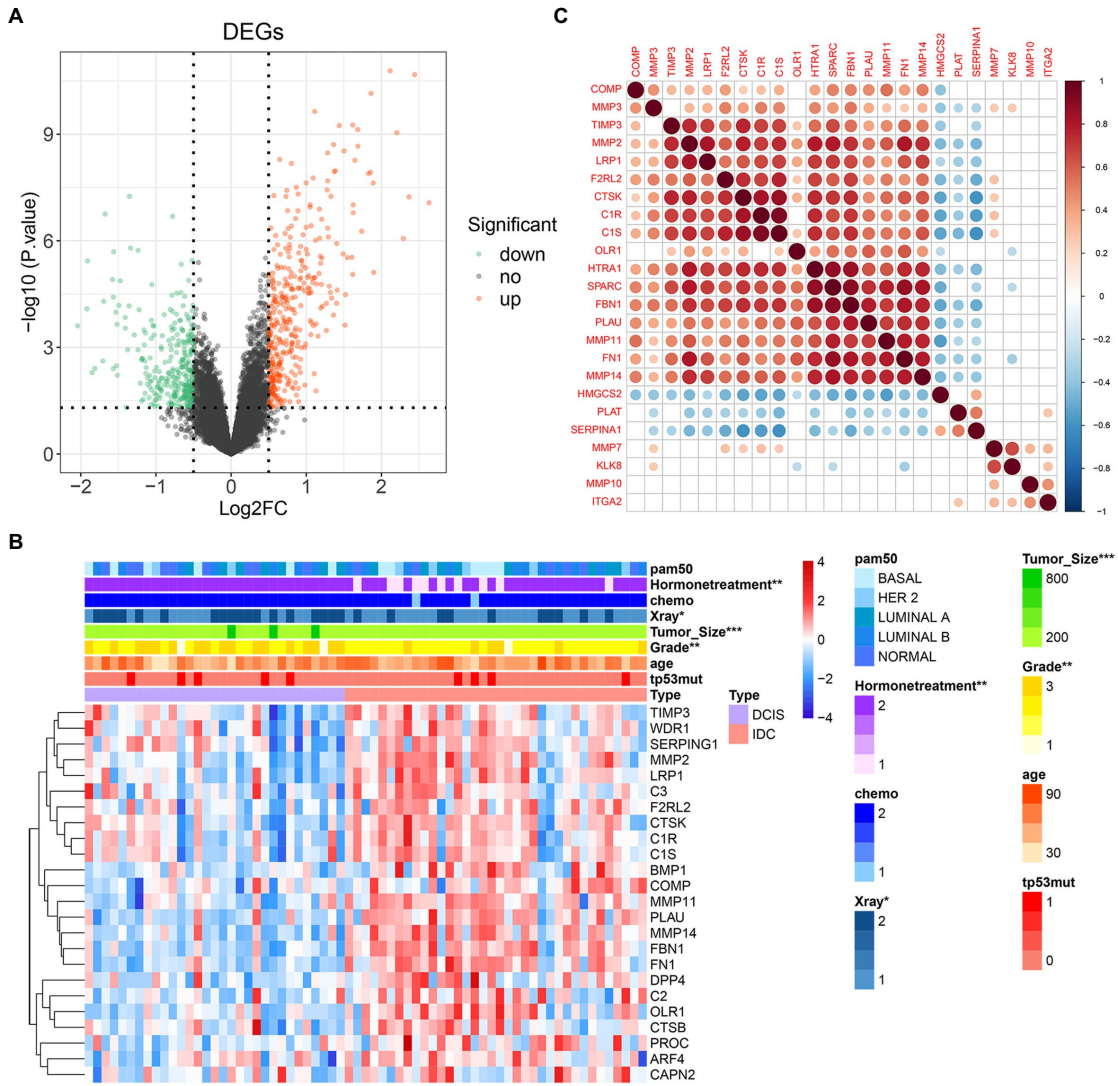
COAGULATION-related genes expression and correlation analysis. **(A)** Volcano map reveals the distribution of downregulated and upregulated DECGs. *X*-axis: fold change; *Y*-axis: −log10 *p*-value. **(B)** Heat map shows the expression of differentially expressed COAGULATION-related genes between DCIS and IDC samples. **(C)** Pearson correlation of these differentially expressed COAGULATION-related genes. ^*^ *p* < 0.05, ^**^ *p* < 0.01, and ^***^ *p* <0.001.

COAGULATION-related genes were extracted from the gene set. The expression levels of differentially expressed COAGULATION-related genes (DECGs) were compared between DCIS and IDC samples from the GSE26304 data set. As shown in Figures 2A,B, a total of 24 DECGs were identified. Pearson correlation analysis showed that there was a significant correlation between the genes, such as *SPARC* was positively correlated with *HTRA1*, *FBN1*, and *FN1*, and *SERPINA1* was negatively correlated with *CTSK*, *C1S*, and *C1R* (Figure 2C).

### Construction of the Prognostic Gene Signature in IDC

**Figure 3:**
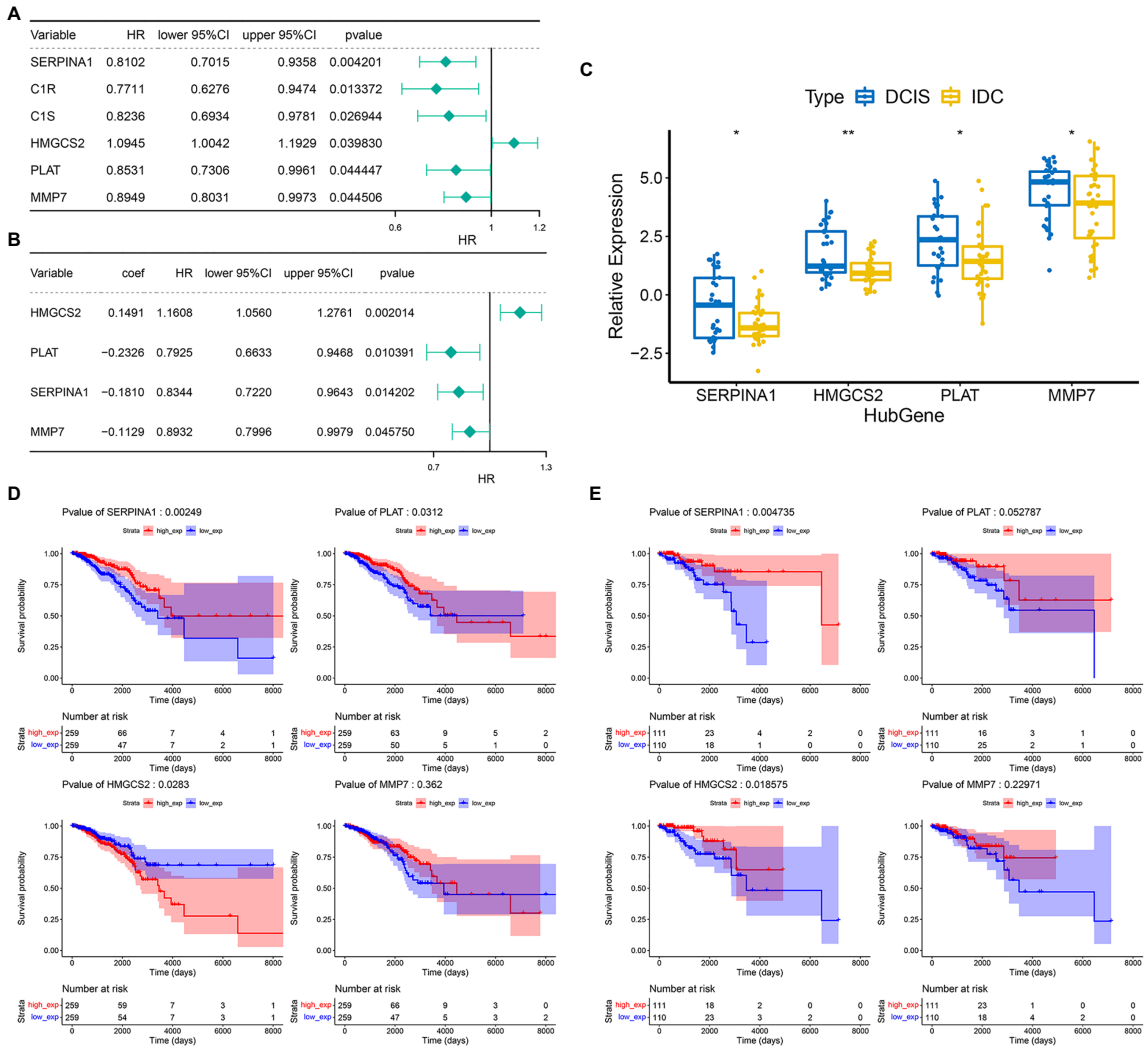
Construction of prognostic gene signature of 4 COAGULATION-related genes in IDC. **(A)** Univariate Cox proportional risk regression forest plot including *p*-values, lower/upper 95%CI, and HR. **(B)** Forest plot for multivariate Cox regression analysis. **(C)** The expression levels of the four prognostic genes between the DCIS and IDC samples in the GSE26304 data set. **(D)** Kaplan–Meier survival curves of model genes in the training set. **(E)** Kaplan–Meier survival curves of model genes in the validation set. ^*^ *p* < 0.05 and ^**^ *p* < 0.01.

We then performed a prognostic analysis of 24 DECGs in the TCGA database. In the present study, 740 IDC patients were randomly divided into training set and validation set according to the 7:3 ratio, and 6 of 24 DEGCs with *p*<0.05 were screened by univariate Cox hazard ratio regression analysis (Figure 3A), which was used to perform a stepwise multivariate Cox proportional hazard analysis. Four genes (*SERPINA1*, *HMGCS2*, *MMP7*, and *PLAT*) were selected as prognostic gene signatures in IDC patients (Figure 3B). The expression levels of the four prognostic genes were significantly downregulated in the IDC patient (Figure 3C). Notably, the expression of *SERPINA1* and *PLAT* significantly correlated with the prognosis of IDC patients, respectively (*p*=0.00249 and 0.0312). However, *HMGCS2* and *MMP7* showed no significant prognostic significance in the IDC patients in the training set (Figure 3D). In addition, while *SERPINA1* and *HMGCS2* demonstrated significant prognostic significance in IDC patients (*p*=0.004735 and 0.018575, respectively), *PLAT* and *MMP7* showed no significant prognostic significance in validation set (Figure 3E).

### Evaluation of the Prognostic COAGULATION-Related Gene Signature

**Figure 4:**
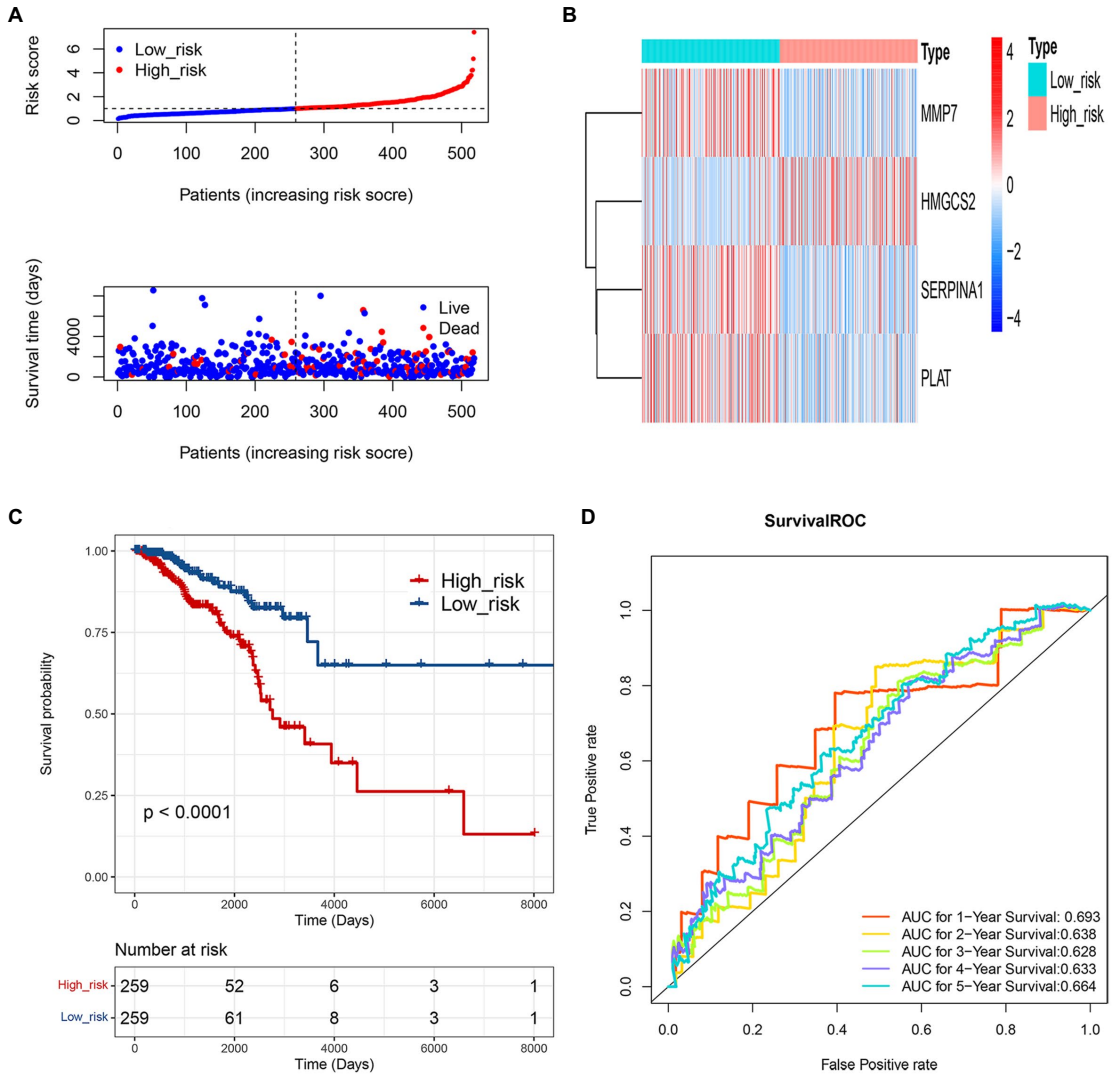
Evaluation of the prognostic gene signature of 4 COAGULATION-related genes. **(A)** Risk curve and its corresponding scatter plots in the training set. **(B)** Heat map of risk model genes in the training set. **(C)** Kaplan–Meier survival curves of high- and low-risk groups in the training set. Red represents high risk, and blue represents low risk. **(D)** Survival ROC curves for 1-, 2-, 3-, 4-, and 5-year survival.

Based on the 4 genes signature, we calculated the risk score for each IDC sample, and then, patients were equally divided into low-risk and high-risk groups in the training set. The risk curve and survival state distribution and risk score heat map in low-risk and high-risk groups were shown in Figures 4A,B. The results of Kaplan–Meier analysis indicated that the survival rate of patients with low-risk score was higher than that of patients with a high-risk score (*p*<0.0001; Figure 4C). Time-dependent ROC analysis showed that the AUC of the COAGULATION-related gene signature was 0.693 for 1year, 0.638 for 2years, 0.628 for 3years, 0.633 for 4years, and 0.664 for 5years, respectively (Figure 4D).

### Validation of the Prognostic COAGULATION-Related Gene Signature

**Figure 5:**
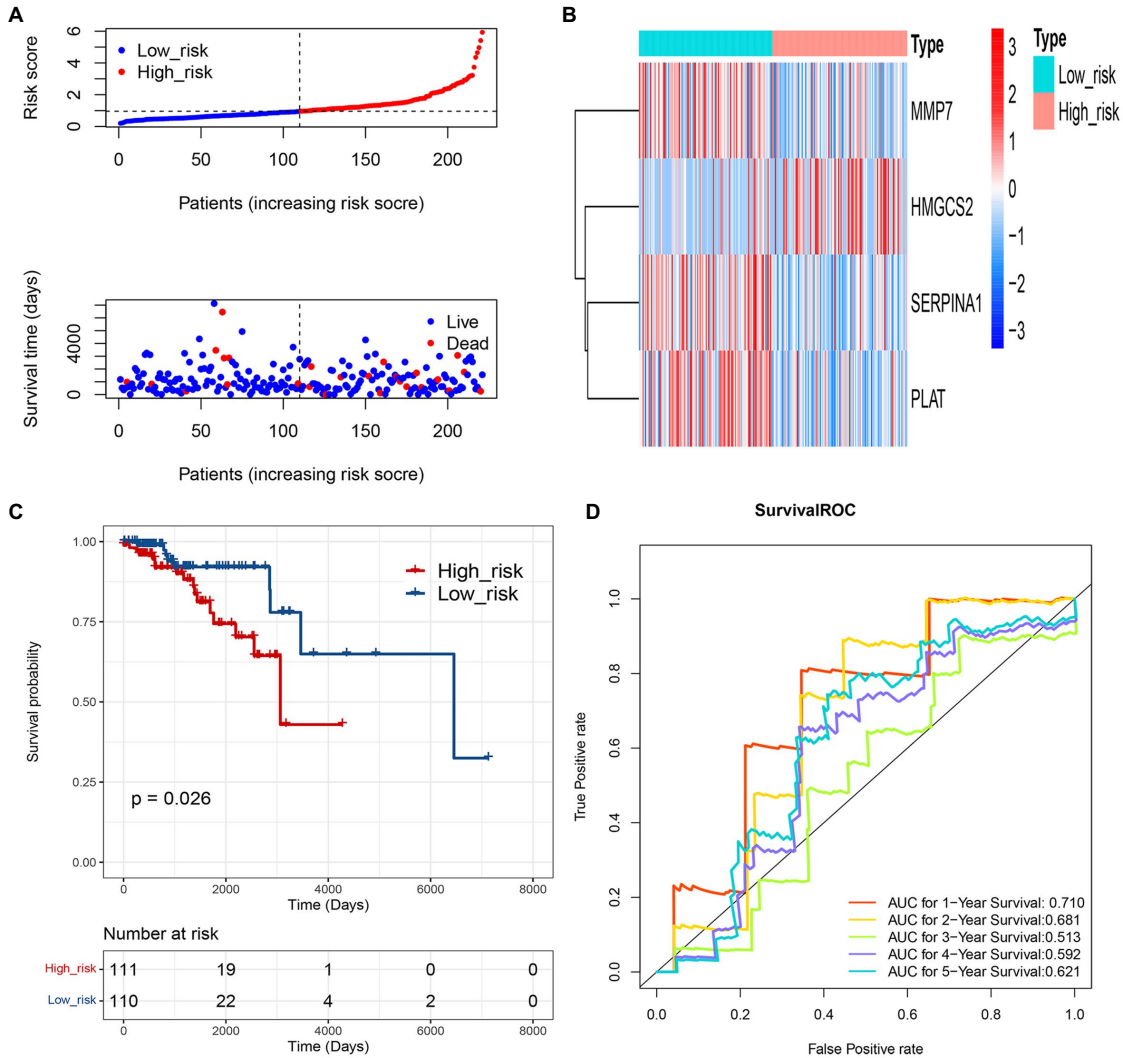
Validation of the prognostic gene signature of 4 COAGULATION-related genes. **(A)** Risk curve and its corresponding scatter plots in the validation set. **(B)** Heat map of risk model genes in the validation set. **(C)** Kaplan–Meier survival curves of high- and low-risk groups in the validation set. Red represents high risk, and blue represents low risk. **(D)** Survival ROC curves for 1-, 2-, 3-, 4-, and 5-year survival.

To further assess the prediction value of this COAGULATION-related gene signature, we constructed risk curve, survival state distribution, overall survival, and ROC curve between low-risk and high-risk groups in the validation set. The risk curve and survival state distribution and risk score heat map were similar to the training set (Figures 5A,B). We observed significantly higher survival rates in the low-risk group than that in the high-risk group in the validation set (Figure 5C). The 1-, 2-, 3-, 4-, and 5-year prognostic accuracies were 0.710, 0.681, 0.513, 0.592, and 0.621, respectively (Figure 5D).

### Evaluation of COAGULATION-Related Gene Signature in the Four Molecular Subtypes of IDC

**Figure 6:**
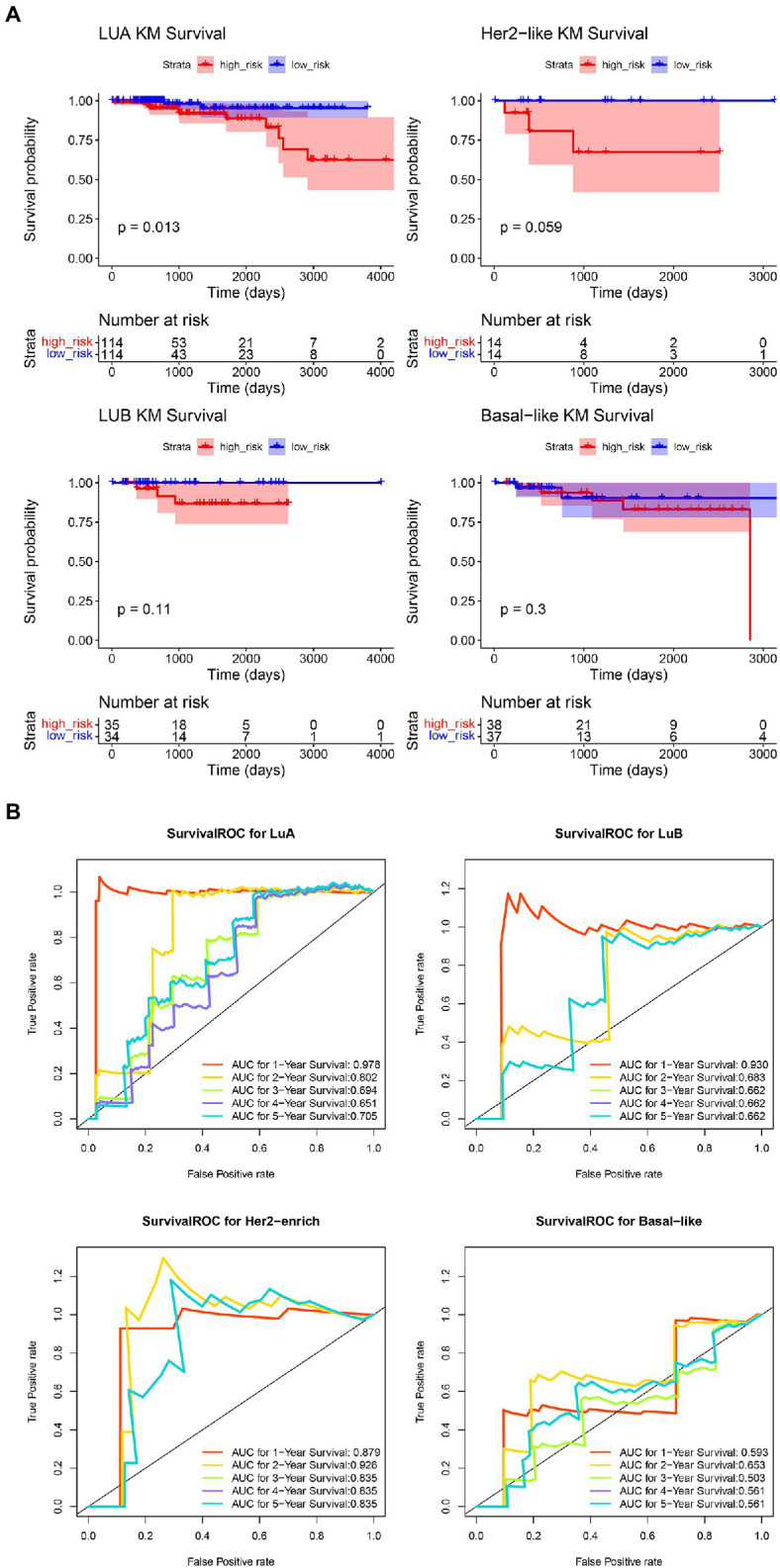
Evaluation of COAGULATION-related gene signature in the four molecular subtypes of IDC. **(A)** The K-M curves of high-risk groups and low-risk groups in the four molecular subtypes of IDC. **(B)** The AUC of ROC curves in predicting 1-, 2-, 3-, 4-, and 5-year OS.

Having shown that the signature based on COAGULATION-related genes can serve as a potent prognostic signature for IDC, we further explored the potential effect of prognosis prediction for the molecular subtypes of IDC (Figure 6). The K-M analysis showed that there was a significant difference in OS between the high-risk groups and low-risk groups of LuA patients (*p*=0.013). The ROC curve analyses at 1-, 2-, 3-, 4-, and 5-year for this prognostic gene signature were conducted to evaluate the predictive efficiency. The results showed that the risk score had high accuracy in predicting the 1- and 2-year survival of LuA patients. However, this signature based on COAGULATION-related genes could not predict the other molecular subtypes, such as LuB, HER2, and basal-like.

### Univariate and Multivariate Cox Regression Analyses of the Independent Prognostic Ability of 4-Gene Signature

**Figure 7:**
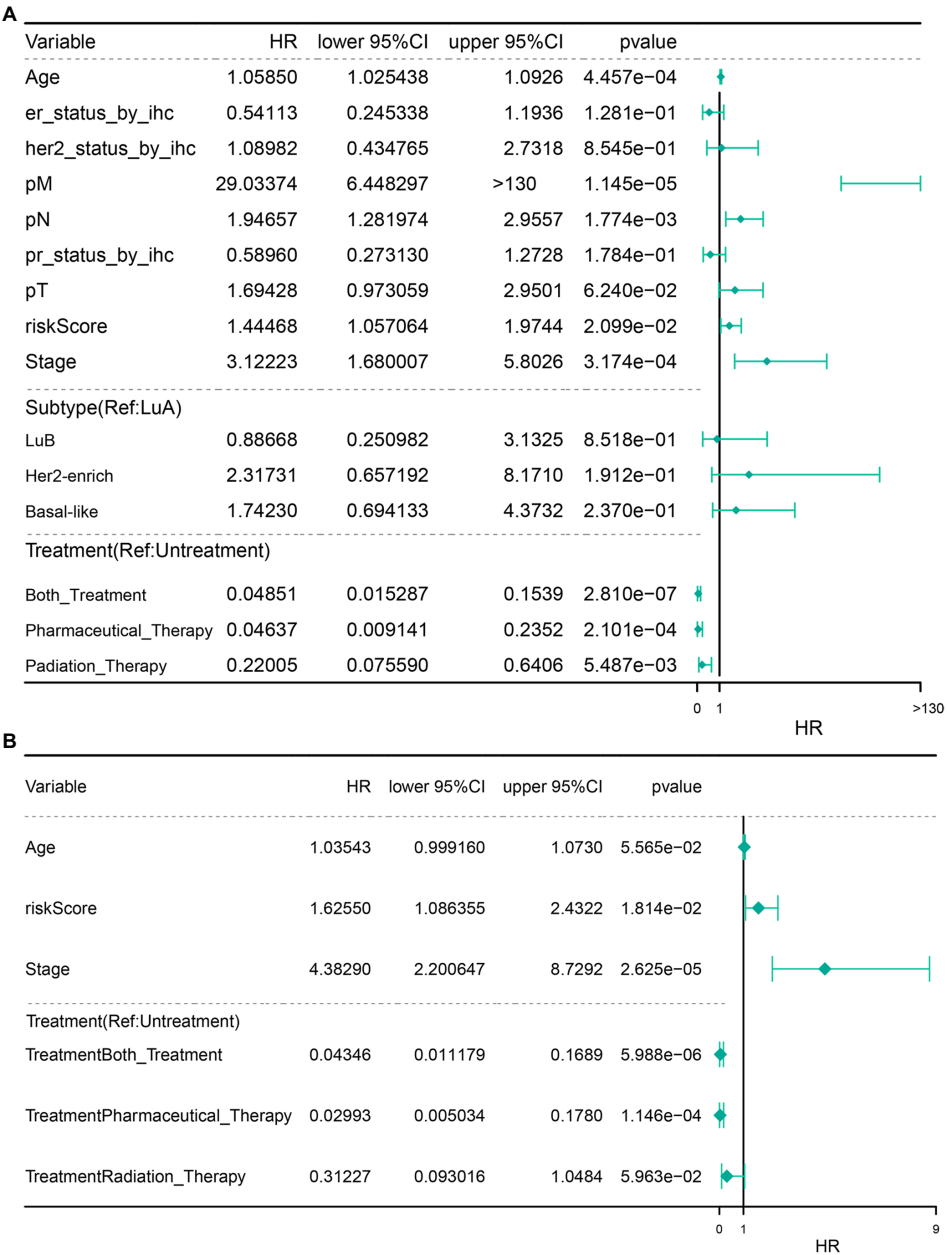
Independent prognostic analysis. **(A)** Independent prognostic univariate Cox regression analysis. **(B)** Independent prognostic multivariate analysis.

Next, we performed univariate and multivariate Cox regression analyses on risk score and other clinical characteristics. The results revealed that the COAGULATION-related gene signature constructed by SERPINA1, HMGCS2, MMP7, and PLAT, pathological_stage, Pharmaceutical_Therapy, and Both_Treatment were the independent prognostic factors that could be used to predict the survival rate of IDC patients (Figures 7A,B).

### Construction of Survival Prediction Nomogram in IDC

**Figure 8:**
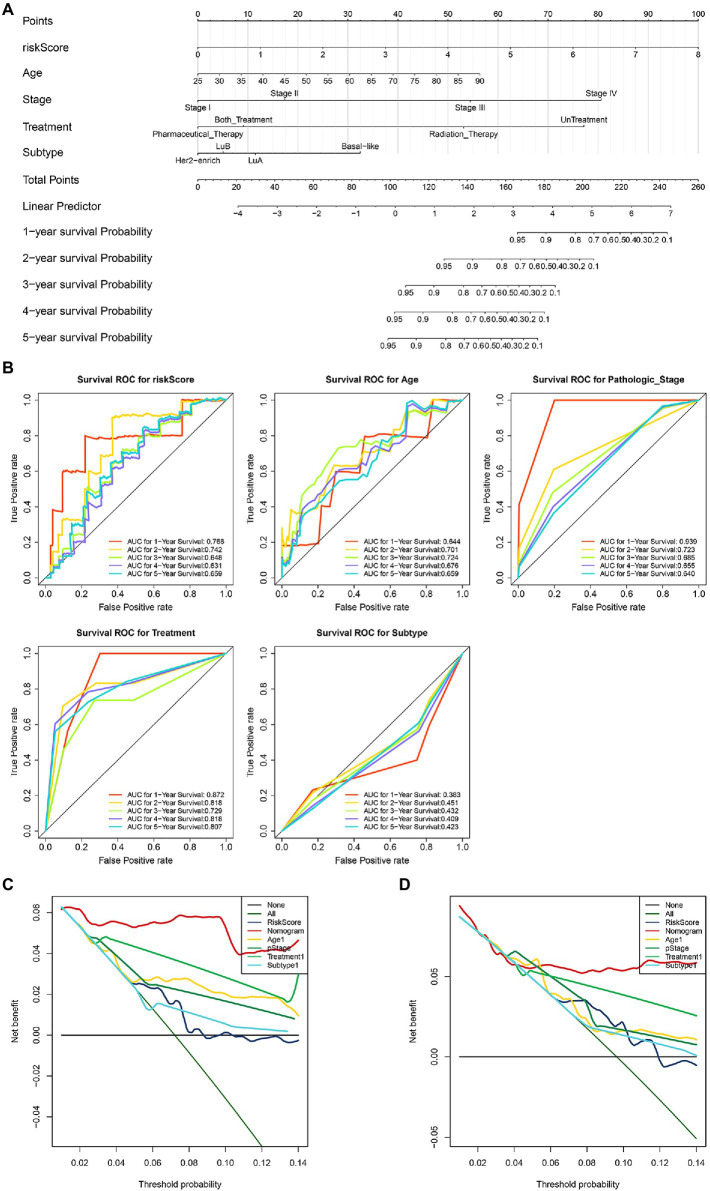
Construction and validation of survival prediction nomogram in IDC. **(A)** A nomogram for predicting the survival rates of 1-, 2-, 3-, 4-, and 5-year. **(B)** ROC of prognostic signature of 4 COAGULATION-related genes, pathological_stage treatment, and molecular subtypes of IDC. **(C)** DCA decision curve for 3-year survival prediction. **(D)** DCA decision curve for 5-year survival predicting.

Based on the independent prognostic factors and molecular subtypes, we constructed a nomogram for predicting 1-, 2-, 3-, 4-, and 5-year survival rates. The nomogram containing pathological_stage, Pharmaceutical_Therapy, Both_Treatment, and molecular subtypes may provide a quantitative method for predicting the 1-, 2-, 3-, 4-, and 5-year survival rate of IDC patients (Figure 8A, c-index=0.82). Every patient got a point for each prognostic parameter, and a higher score indicated a worse prognosis. ROC analyses indicated that the prognostic accuracy of the COAGULATION-related gene signature-based prognostic nomogram was compared with those of pathological_stage treatment and molecular subtypes of IDC (Figure 8B). The results of 3- and 5-year DCA demonstrated that the independent prognostic factors had a high potential for clinical application (Figures 8C,D).

### Validation of Prognostic Signature Genes Expression in IDC/DCIS Tissues

**Figure 9:**
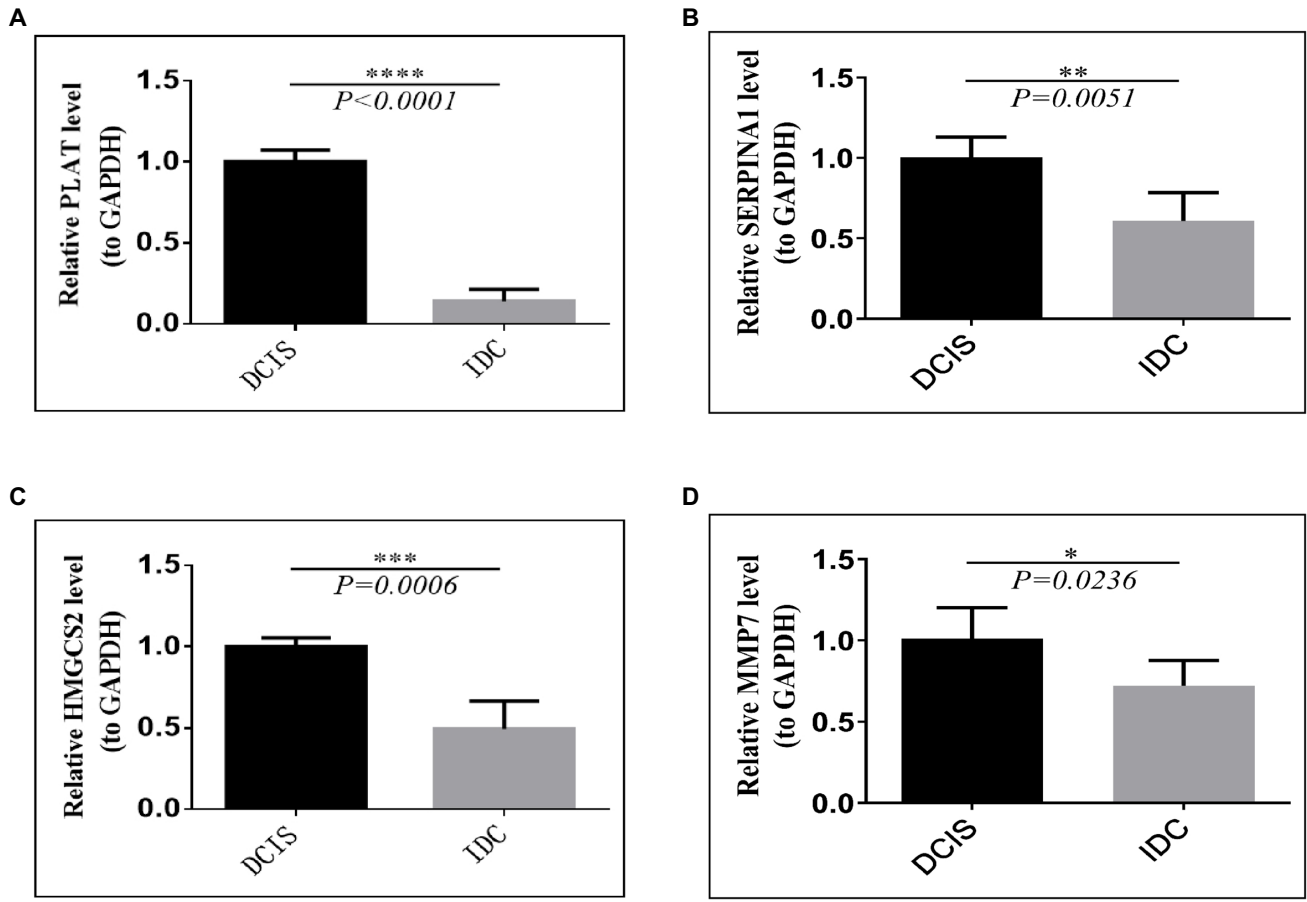
Validation of prognostic signature gene expression in IDC/DCIS tissues. The mRNA expression patterns of four prognostic between DCIS and IDC tissues. **(A)** PLAT**, (B)** SERPINA1**, (C)** HMGCS2, and **(D)** MMP7. All experimental data were expressed in means ± SD (IDC: *n*=10, DCIS: *n*=5). ^*^ *p*<0.05, ^**^ *p*<0.01, ^***^ *p*<0.001, ^****^ vs. the DCIS group.

To assess the genes that are significant for the construction of the risk score signature, we performed the qRT-PCR assay. The results of the qRT-PCR analysis demonstrated that *PLAT*, *SERPINA1*, *HMGCS2*, and *MMP7* were significantly downregulated in IDC tissues, compared with the DCIS tissues (all *p*<0.05, Figures 9A–D), which were consistent with the results obtained from the GSE26304 data set.

## Discussion

The incidence of breast cancer is increasing year by year, and despite the improvement of treatments, it is very difficult to treat in the event of recurrence or metastasis. Therefore, identifying effective prognostic biomarkers is an indispensable step in predicting breast cancer patients’ disease and controlling the progress of treatment. With the rapid development of molecular genomics, highly specific diagnostic and prognostic biomarkers are providing molecular evidence to improve conventional diagnostic and therapeutic options.

In this study, we first found that the “HALLMARK_COAGULATION” pathway was activated in IDC based on the results of GSVA analysis. The enrichment of the most COAGULATION genes in IDC indicates a potential role of these genes in the tumorigenesis and development of IDC. There were studies reporting found that patients with IDC had higher DD levels, hypercoagulation, secondary fibrinolysis, and increased fibrin degradation (Bazzi et al., 2016; Dai et al., 2018; Shaker et al., 2020). There is evidence that fibrin formed by fibrinogen decomposition not only provides scaffolding for tumor cells but also acts as a ligand to promote adhesion binding between platelets and tumor cells, thus promoting the infiltration and metastasis of tumor cells (Mego et al., 2015). Secondly, patients with IDC have high PLT aggregation activity and low erythrocyte count, which may increase erythrocyte aggregation and plasma viscosity, resulting in impaired oxygen transport efficiency and microcirculatory hypoxia, which is conducive to tumor sedimentation, metastasis, and thrombosis (Tikhomirova et al., 2016). In a hypercoagulable state, tumor cells can interact with PLT immediately (within 2min) after leaving the primary site and entering the bloodstream, and PLT aggregates and secretes VEGF, which increases the local vascular density of the tumor (Krenn-Pilko et al., 2015), immediately surrounding the tumor cells and protecting them from blood impact and NK cell-based immune responses (Placke et al., 2011). In addition, PLT induces EMT in tumor cells (Labelle et al., 2011), giving the cells the characteristics of tumor stem cells with increased malignancy (Scheel et al., 2011; Ye and Weinberg, 2015). It also contributes to the exudation of tumor cells from the blood by secreting chemokines CXCL5 and CXCL7 that activate the granulocyte expression receptor CXCR2. Therefore, in the infiltration and angiogenesis of breast malignancies, tumor development forms a vicious circle with the activation of coagulation-related factors and cells.

We used transcriptomic data and clinical data of IDC patients in the TCGA database to construct a prognostic model of four genes (*SERPINA1*, *HMGCS2*, *MMP7*, and *PLAT*) related to the coagulation pathway. We further verified the actual expression of these four genes in the GSE26304 clinical samples using PCR technique. Among them, *SERPINA1* and *PLAT* showed significant correlations. SERPINA1, a plasma protein, is synthesized mainly in hepatocytes, which is mainly involved in angiogenesis, intravascular fibrinolysis, wound healing, tumor invasion, and metastasis. It has been reported that the expression of SERPINA1 was correlated with the prognosis of metastasis in a variety of cancers, including lung, colon, and bladder cancers (Zhang et al., 2014; Kwon et al., 2015; Ercetin et al., 2019). Our study showed that *SERPINA1* is a potential protective gene expressed in breast cancer, which is consistent with previous studies (Chan et al., 2015). *PLAT* is a gene encoding tissue-type plasminogen activator (tPA), which is synthesized and secreted mainly by vascular endothelial cells. PLAT has a role in promoting fibrinolysis and antagonizing thrombosis and is likely involved in tumor oncogenesis and progression (Jia et al., 2019). *HMGCS2* is localized at 1p12, which encodes a mitochondrial hydroxymethylglutaryl coenzyme A synthase (HMGCS) found mainly in the liver and testicular tissues (Hu et al., 2017). It is the first rate-limiting enzyme in ketone body synthesis and can drive malignancy progression and metastasis by promoting ketone body production and reuse (Koutnik et al., 2020; Wang et al., 2020). MMP-7 is a stromal lysin protein, and many studies have shown that MMP-7 plays a significant regulatory role in apoptosis, immunity, cell migration, and angiogenesis pathways. The function of MMP7 usually complements the classical tumor properties, leading to invasion, immune immunity, and metastasis of tumor cells and plays an important role in the carcinogenesis process (Jobin et al., 2017). Many malignant tumors including gastrointestinal tumors, breast cancer, and head and neck tumors are found to have a high expression of MMP-7 (Gobin et al., 2019; Dirks et al., 2020).

This study, combining with clinical features, demonstrated that pathological_stage, Pharmaceutical_Therapy, Both_Treatment, and molecular subtypes have independent prognostic value, and based on this finding, we constructed a nomogram to predict IDC survival from 1 to 5years, which may provide a platform for clinical assessment of breast cancer. However, further studies with more clinical samples and information are needed to test the benefits and value of this prognostic model.

## Conclusion

Overall, through analyzing the “HALLMARK_COAGULATION” pathway in IDC and its potential prognostic value in IDC, we constructed a COAGULATION-related gene-based prognostic model and developed a nomogram for predicting the survival of IDC patients within 1–5years. In this model, the expression of SERPINA1 and PLAT was significantly correlated with the survival of IDC. Thus, we propose that the expressions of SERPINA1 and PLAT warrant further investigation for their potential as new predictive biomarkers for IDC.

## Data Availability Statement

The datasets presented in this study can be found in online repositories. The names of the repository/repositories and accession number(s) can be found in the article/supplementary material.

## Author Contributions

All authors participated in the design of this study and read and approved the final version of the manuscript. JD was in charge of data obtainment and analysis. JL was in charge of statistical analysis and drafted the manuscript. YW and HJ supervised this study and revised the manuscript.

## Funding

The research fund was provided by General Project of Natural Science Foundation of Shanxi Province (201901D111347).

## Conflict of Interest

The authors declare that the research was conducted in the absence of any commercial or financial relationships that could be construed as a potential conflict of interest.

## Publisher’s Note

All claims expressed in this article are solely those of the authors and do not necessarily represent those of their affiliated organizations, or those of the publisher, the editors and the reviewers. Any product that may be evaluated in this article, or claim that may be made by its manufacturer, is not guaranteed or endorsed by the publisher.

## Footnotes

^1^ https://www.ncbi.nlm.nih.gov/geo/

^2^ https://portal.gdc.cancer.gov/

^3^ https://CRAN.R-project.org/package=pheatmap

^4^ https://CRAN.R-project.org/package=forestplot

^5^ https://CRAN.R-project.org/package=survminer

^6^ https://CRAN.R-project.org/package=rms
